# SPyCi-PDB: A modular command-line interface for back-calculating experimental datatypes of protein structures

**DOI:** 10.21105/joss.04861

**Published:** 2023-05-10

**Authors:** Zi Hao Liu, Oufan Zhang, João M. C. Teixeira, Jie Li, Teresa Head-Gordon, Julie D. Forman-Kay

**Affiliations:** 1 Molecular Medicine Program, Hospital for Sick Children, Toronto, Ontario M5G 0A4, Canada; 2 Department of Biochemistry, University of Toronto, Toronto, Ontario, M5S 1A8, Canada; 3 Pitzer Center for Theoretical Chemistry, University of California, Berkeley, California 94720-1460, USA; 4 Department of Chemistry, University of California, Berkeley, California 94720-1460, USA; 5 Department of Biomedical Sciences, University of Padova, Padova 35131, Italy; 6 Department of Chemical and Biomolecular Engineering, University of California, Berkeley, California 94720-1462, USA; 7 Department of Bioengineering, University of California, Berkeley, California 94720-1762, USA

## Summary

Structural determination of proteins has been a central scientific focus since the early 1960s (Dill et al., 2008) with technological advances facilitating experimental structures of stable, folded proteins by nuclear magnetic resonance (NMR) spectroscopy (Kanelis et al., 2001), X-ray crystallography (Smyth, 2000), and cryo-electron microscopy (Malhotra et al., 2019), as well as the recent computational prediction of structures (Baek et al., 2021; Jumper et al., 2021). Modeling intrinsically disordered proteins (IDPs) and intrinsically disordered regions (IDRs), however, remains challenging due to their highly dynamic nature and low propensity to form low energy folded structures (Mittag & Forman-Kay, 2007).

Currently, approaches to model IDPs/IDRs generally start with initial pools of structures that sample potentially accessible conformations and then utilize experimental data to narrow the pool. One method to generate initial conformational ensembles of IDPs/IDRs uses sampling techniques such as in TraDES (Feldman & Hogue, 2000, 2001), Flexible-meccano (Ozenne et al., 2012), FastFloppyTail (Ferrie & Petersson, 2020), IDPConformerGenerator (Teixeira et al., 2022), and others (Estaña et al., 2019), that rely on the torsion angle distributions found in high-resolution folded protein structures deposited in the RCSB Protein Data Bank (Berman, 2000). Another more computationally expensive approach generates conformational ensembles using molecular dynamics (MD) simulations with different force-fields (Robustelli et al., 2018; Salvi et al., 2016).

After generating the initial pool of structures, back-calculations to experimental data and reweighting using Monte-Carlo (Krzeminski et al., 2012) or Bayesian statistics (Bottaro et al., 2020; Brookes & Head-Gordon, 2016; Lincoff et al., 2020) can be performed to define structural ensembles that better match solution NMR, small-angle X-ray scattering (SAXS), single molecule fluorescence (SMF), and other experimentally obtained data from these IDPs/IDRs. An emerging method to generate conformations of IDPs/IDRs uses machine learning generative models based on ensembles generated from sampling or MD techniques as training data and reinforces learning with experimental data (Zhang et al., 2022). Both of these general approaches rely on back-calculation of “experimental observables” from coordinates of conformers within the ensembles, a task that is increasingly complex due to the various models for interpretation of experimental data and the numerous tools available.

Here we present **SPyCi-PDB**, designed to facilitate and streamline this back-calculation stage by acting as a platform for internal back-calculator functions as well as published third-party software, utilizing PDB structures of disordered protein conformations. One goal of **SPyCi-PDB** is to minimize the existing issues with different data-formats from software and scripts within the IDP/IDR research community and improve accessibility to researchers with a range of computational expertise. In this release, **SPyCi-PDB** can back-calculate NMR chemical shift (CS), paramagnetic resonance enhancement (PRE), nuclear Overhauser effect (NOE), 3J-HNHA coupling (JC), and residual dipolar coupling (RDC) data; hydrodynamic radius (Rh) data from NMR, light scattering, or size exclusion chromatography; SAXS; and single-molecule fluorescence resonance energy transfer (smFRET) values from all-atom PDB structures of IDP/IDR conformations.

## Statement of Need

As new software packages and *in silico* methodologies emerge to better model IDP/IDR structures, back-calculations to multiple experimental datatypes are required to quantitatively assess the conformers generated. However, interpretation of solution data as a simple calculation from the sum of sampled conformations within IDP/IDR ensembles is fraught with pitfalls. For example, commonly used approaches for back-calculating NOE and PRE data for dynamic protein systems treat only the distance and do not incorporate the contribution of dynamics of the vector connecting the interacting points, potentially leading to underestimations of the potential range of distances sampled (Brookes & Head-Gordon, 2016; Krzeminski et al., 2012; Lincoff et al., 2020). In addition, even for stable systems, back-calculation is not trivial, with even state-of-the-art back-calculators of chemical shifts, such as in UCBShift (Li et al., 2020), leading to errors that can be large relative to the expected deviation of experimental values. Given the rapidly developing nature of different software tools to perform back-calculations, **SPyCi-PDB** should assist by providing a user-friendly, all-in-one package to reduce time and confusion in this back-calculation step as well as open opportunities for future collaborations and integration of new experimental datatypes. Furthermore, **SPyCi-PDB** aims to unify different input and output data formats from different experimental datatypes to increase productivity and accelerate research. As stated in the documentation hosted by ReadTheDocs, input formats are conventional comma-delimited tables (e.g. .CSV, .TXT), while the output format is human-readable .JSON files that can be easily manipulated using Python or other software based on the user’s ultimate needs. **SPyCi-PDB** was also developed to integrate into the IDPConformerGenerator platform (Teixeira et al., 2022).

Ultimately, given the complicated and dynamic exchanging nature of IDPs, new back-calculators are needed to be developed to address the current challenges in interpretation. By creating a tool with modularity and best practices, we aim to encourage the researcher community to contribute towards this platform to further the goal of improved modelling of IDPs and IDRs.

## Implementation

As **spycipdb** is written completely in Python, it is compatible with any platform able to execute Python (>=3.8, <4.0). However, certain third-party extensions to perform back-calculations (SAXS and RDC) have only been tested on 64-bit Ubuntu 18.04.X LTS and 20.04.X LTS, as well as WSL 2.0 on 64-bit Windows 11.

In the production version 0.3.X, four out of eight modules of **SPyCi-PDB**’s back-calculators (pre, noe, jc, smfret) use internal mathematical equations and PDB structure processing algorithms from IDPConformerGenerator libraries (Teixeira et al., 2022). The pre (1) and noe (2) module calculates scalar distances between pairs of atoms according to the pairs derived from the experimental template. It utilizes an algorithm that matches atom names of each residue with allowance for multiple assignments for noe. The jc (3) module uses the Karplus curve, a simple cosine function, to back-calculate the desired J-couplings according to residue number as provided by the experimental template file (Pérez et al., 2001). Finally, the smfret (4) module takes into consideration residue pairs and a scale factor to adjust for dye size from the experimental setup to back-calculate distances between two alpha-Carbon (CA) atoms (Lincoff et al., 2020). The aforementioned equations are as follows:

(1) $$\sqrt{\delta x^{2}+\delta y^{2}+\delta z^{2}}$$

(2) $$\sqrt[{6}]{(\frac{{\left(\delta x^{2}+\delta y^{2}+\delta z^{2}\right)}^{3}}{N}}$$

(3) $$\text{cos}\left(\varphi -\frac{\pi }{3}\right)$$

(4) $$\frac{1}{1+{\left(\frac{D\cdot \sqrt{\frac{\left|R_{1}-R_{2}\right|+7}{R_{1}-R_{2}}}}{S}\right)}^{6}}$$

Where δx, δy, δz are the cartesian differences between two atoms of interest (1, 2), N represents the number of combinations for NOE atom pairs (2), φ is the Phi torsion angle of interest (3), D is the scalar distance between the residues of interest with R1 and R2 being the vector cartesian co-ordinates for the residues and S being the scale factor according to experimental information.

The remaining 4 modules (cs, saxs, rh, rdc) call upon third-party academic software: UCBShift, a machine learning algorithm that uses structural alignment for experimental chemical shift replication and employs a random forest regression on curated data to most accurately predict protein chemical shifts (Li et al., 2020); CRYSOL v3, an updated version of the well-established SAXS back-calculator from ATSAS that can now evaluate the hydration shell by populating the protein structure with dummy water (Franke et al., 2017); HullRad, to calculate hydrodynamic radius (Rh) by using a convex hull model to estimate the hydrodynamic properties of a macromolecule (Fleming & Fleming, 2018); and PALES, using the steric obstruction model to derive dipolar coupling (RDC) information from the average orientation of the 3D coordinates (Zweckstetter & Bax, 2000). Thorough testing of each module has been performed to ensure smooth installation and troubleshooting as well as retaining or providing multiprocessing capabilities that may not have been implemented in their standalone forms. When choosing third-party software, we prioritized those written in Python for ease of integration.

We plan to integrate alternative methods to calculate experimental datatypes internally such as using a parameterizable fluorescence lifetime and the Förster distance, as used in the Naudi-Fabra et al. study of describing intrinsically disordered proteins using smFRET, NMR, and SAXS (Naudi-Fabra et al., 2021). Future additions to the **SPyCi-PDB** interface suite are welcome and easy to perform given its modular design.

**Figure 1:**
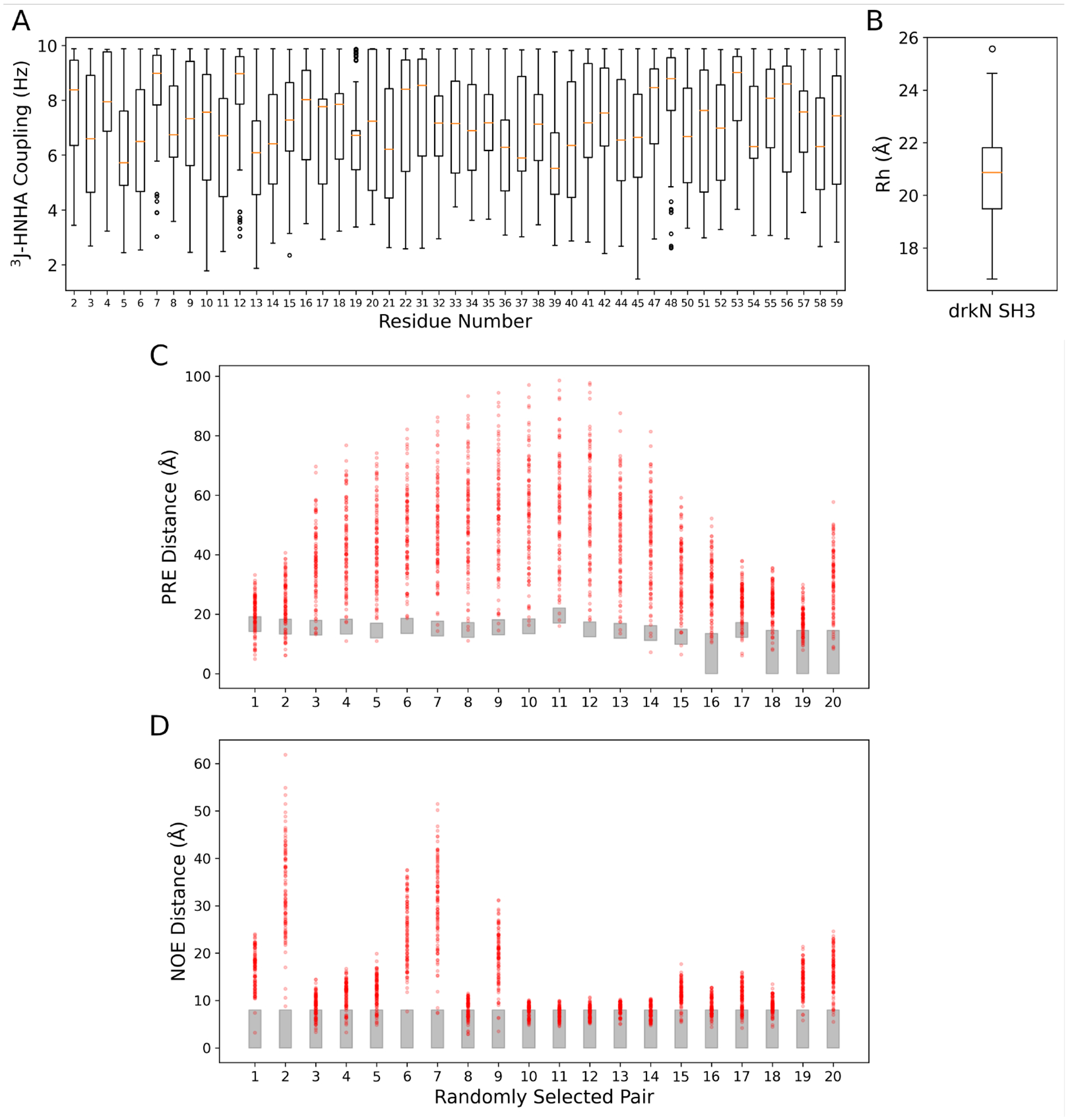
Plots of distributions of back-calculated experimental data of 100 structures of the unfolded state of the Drk N-terminal SH3 domain (drkN SH3) generated using IDPConformerGenerator (Teixeira et al., 2022). Panel (A) shows back-calculated 3J-HNHA couplings in Hz based on the Karplus equation with A, B, and C constants from Lincoff et al. (Lincoff et al., 2020). Only residues with experimental data to compare will generate a back-calculated J coupling value. Panel (B) shows the distribution of back-calculated Rh values in Angstroms using HullRad (Fleming & Fleming, 2018). Panels (C) and (D) show twenty randomly selected pairs of back-calculated PRE and NOE distances, respectively. The ranges of experimental values are represented as grey boxes while back-calculated values for each conformer are shown as red dots.

Detailed installation/troubleshooting instructions, real-world usage examples, and input/output formats are provided both in the project’s documentation hosted on ReadTheDocs (https://spyci-pdb.readthedocs.io/en/stable/) and within the modules through the --help argument. Plots of sample outputs from the jc, rh, pre, and noe modules using the example structures and data in the repository are shown in Figure 1.

Comparing the back-calculated PRE and NOE distance values to the experimental observables, the default Euclidean distance interpretation yields some values agreeing with the experimental range (Figure 1C, 1D). With a greater sample size, we would likely capture more back-calculated data agreeing with the experimental ranges. The internal plotting features in the noe, pre, rh, and jc modules of **SPyCi-PDB** is useful for users to gauge the quality of the initial pool before downstream reweighting. Furthermore, with the integration of different back-calculation methods, these plots will provide the user with a useful comparison between back-calculation philosophies.
